# Monitoring and managing metabolic effects of antipsychotics: a cluster randomized trial of an intervention combining evidence-based quality improvement and external facilitation

**DOI:** 10.1186/1748-5908-8-120

**Published:** 2013-10-08

**Authors:** Richard R Owen, Karen L Drummond, Kristen M Viverito, Kathy Marchant, Sandra K Pope, Jeffrey L Smith, Reid D Landes

**Affiliations:** 1 VA Center for Mental Healthcare & Outcomes Research, Central Arkansas Veterans Healthcare System, 2200 Fort Roots Dr., North Little Rock, AR, USA; 2 Department of Psychiatry, University of Arkansas for Medical Sciences College of Medicine, 4301 West Markham, Little Rock, AR, USA; 3 VA Mental Health Quality Enhancement Research Initiative (MH QUERI), Central Arkansas Veterans Healthcare System, 2200 Fort Roots Dr., North Little Rock, AR, USA; 4 Department of Biostatistics, University of Arkansas for Medical Sciences College of Medicine, 4301 West Markham, Little Rock, AR, USA

**Keywords:** Evidence-based quality improvement, External facilitation, Metabolic side effects, Metabolic monitoring, Metabolic management, Antipsychotic side effects, Veterans, Mental health

## Abstract

**Background:**

Treatment of psychotic disorders consists primarily of second generation antipsychotics, which are associated with metabolic side effects such as overweight/obesity, diabetes, and dyslipidemia. Evidence-based clinical practice guidelines recommend timely assessment and management of these conditions; however, research studies show deficits and delays in metabolic monitoring and management for these patients. This protocol article describes the project ‘Monitoring and Management for Metabolic Side Effects of Antipsychotics,’ which is testing an approach to implement recommendations for these practices.

**Methods/Design:**

This project employs a cluster randomized clinical trial design to test effectiveness of an evidence-based quality improvement plus facilitation intervention. Eligible study sites were VA Medical Centers with ≥300 patients started on a new antipsychotic prescription in a six-month period. A total of 12 sites, matched in pairs based on scores on an organizational practice survey, were then randomized within pairs to intervention or control conditions.

Study participants include VA employees involved in metabolic monitoring and management of patients treated with antipsychotics at participating sites. The intervention involves researchers partnering with clinical stakeholders to facilitate tailoring of local implementation strategies to address barriers to metabolic side-effect monitoring and management. The intervention includes a Design Phase (initial site visit and subsequent development of a local implementation plan); Implementation Phase (guided by an experienced external facilitator); and a Sustainability Phase. Evaluation includes developmental, implementation-focused, progress-focused and interpretative formative evaluation components, as well as summative evaluation. Evaluation methods include surveys, qualitative data collection from provider participants, and quantitative data analysis of data for all patients prescribed a new antipsychotic medication at a study site who are due for monitoring or management of metabolic side effects during the study phases. Changes in rates of recommended monitoring and management actions at intervention and control sites will be compared using time series analyses.

**Discussion:**

Improving monitoring for metabolic side effects of antipsychotics, as well as promoting timely evidence-based management when these effects emerge, will lead to improved patient safety and long-term outcomes. This article discusses key strengths and challenges of the study.

**Trial registration:**

NCT01875861

## Background

Psychotic disorders are prevalent, disabling and costly among Veterans receiving healthcare in the U.S. Department of Veterans Affairs (VA) and elsewhere. For example, while 3.4% of VA service users have a diagnosis of schizophrenia, patients with this serious mental illness (SMI) account for 11.7% of VA healthcare costs [1]. In Fiscal Year (FY) 2010, about 242,000 patients with a psychotic disorder diagnosis (*e.g*., schizophrenia, bipolar disorder) were treated in the VA [2]. Over 90% of these patients who are treated with antipsychotic medication are prescribed a second-generation antipsychotic (SGA) medication. Unfortunately, as was recently reinforced by the landmark Clinical Antipsychotic Trials of Intervention Effectiveness (CATIE) study [3], treatment with many SGAs is associated with metabolic side effects such as overweight/obesity, diabetes, and dyslipidemia [4-7]. Failure to properly monitor and manage these side effects can lead to increased risk of mortality due to diabetic ketoacidosis [8] and cardiovascular disease [9]. In addition to these treatment-emergent adverse effects, patients with SMIs such as schizophrenia already have a greater prevalence of obesity (42%) [10,11] and diabetes (13%) [12] than the general population.

### Recommendations for monitoring and management of metabolic side effects

A national conference organized in 2002 by the VA Mental Health Quality Enhancement Research Initiative (MH QUERI) and others developed evidence- and expert consensus-based recommendations for metabolic side-effect monitoring [13]. Subsequently, similar recommendations were developed by a joint panel of the American Diabetes Association (ADA) and the American Psychiatric Association (APA) [14]. Monitoring recommendations from both of these sources were incorporated in the 2004 VA/Department of Defense (DoD) Psychoses Guidelines update [15], and relevant evidence-based management recommendations are contained in VA clinical practice guidelines (CPG) for obesity [16], diabetes [17] and dyslipidemia [18]. Briefly, monitoring of weight or body mass index (BMI), fasting plasma glucose (FPG), and fasting plasma lipids is recommended when a patient is started on a new antipsychotic medication, with monthly follow-up monitoring of weight/BMI, and FPG and lipid profile monitoring at three to four months and annually thereafter [13,14]. In addition, the ADA/APA panel suggested using SGAs with lower propensity for weight gain. Ziprasidone and aripiprazole are least likely to cause weight gain, while olanzapine and clozapine are the most likely [14]. Another recommendation from the CPG for Screening and Management of Overweight and Obesity [16] provides strong evidence-based recommendations that overweight or obese patients be offered weight loss interventions that combine dietary therapy, increased physical activity, and behavioral modification strategies. Implementation of the overweight/obesity CPG is specifically supported by the ‘MOVE!’ Weight Management Program for Veterans, a national VA-designed program to help Veterans lose weight, keep it off, and improve their health [19].

### Existing VA practice, patterns and outcomes

Both VA [7,20-22] and non-VA [23-25] studies have found low rates of metabolic monitoring among patients treated with antipsychotics. Surprisingly, there are no published studies that examine the rates for management of metabolic side-effects that develop in the context of newly-initiated antipsychotic treatment or antipsychotic medication changes, although substantial improvement is likely needed in these practices as well. In a recent study of 32 VA facilities, metabolic monitoring frequency was greater at baseline (*i.e*., within 30 days before to 30 days after the new prescription date) than at initial follow-up (*i.e*., 60 to 120 days after prescription date), although at both times monitoring was low. For example, baseline monitoring rates compared to follow-up rates were 67% versus 49% for weight, 46% versus 27% for glucose or hemoglobin A1c, and 32% versus 16% for LDL [20].

### Preliminary studies and related developments

A previous project, ‘A Study of Strategies for Improving Schizophrenia Treatment (ASSIST),’ compared the effectiveness of two strategies aimed at improving medication management for schizophrenia – a team-based quality improvement strategy (Team QI) and a strategy involving a single clinical opinion leader (OL) at six sites in two Veterans Integrated Service Networks (VISNs). Both strategies were aided by an evidence-based QI (EBQI) approach and external facilitation (EBQI/F). While both Team QI and OL strategies resulted in improvements in metabolic monitoring rates at the time of a new antipsychotic prescription, neither strategy was clearly superior to the other. The study suggested that: designating a provider or clinic to ensure completion of monitoring, and implementing a computer routine that queried the VA electronic medical record to identify patients due for monitoring were effective QI components.

Subsequently, in December 2007, the VA Office of Inspector General (OIG) reported on their examination of metabolic monitoring and management for patients on atypical antipsychotics for ≥90 days [26]. Rates of monitoring described in the OIG report were higher than in other studies (*e.g*., 88% of patients had an FPG test in the past year) because of longer observation periods than in other studies. For obese or overweight patients (82% of the sample), 21% did not have any record of a weight management intervention. Of the 17% of patients with elevated glucose in the past three years, only 49% had documented management. A limitation of this study was that it looked at any metabolic management within the past year, rather than examining timely intervention for abnormal monitoring results. The OIG report and VA research clearly demonstrate the need for more timely guideline-concordant management of antipsychotics’ metabolic side effects.

Also in 2007, MH QUERI and the South Central Mental Illness Research, Education and Clinical Center (MIRECC) sponsored an expert panel meeting to further operationalize metabolic monitoring recommendations and consider applicability of the other VA/DoD CPG recommendations for management of metabolic side effects. Panel discussions informed a subsequent task force, the Atypical Antipsychotic Workgroup, formed by VA Mental Health Services in 2008. Both groups, after review of existing guidelines and relevant scientific literature, noted the importance of broad dissemination of evidence-based and expert consensus-based recommendations for monitoring and managing antipsychotic side effects.

### MIRECC initiative on antipsychotic monitoring improvement project

Following the Atypical Antipsychotic Workgroup report, Mental Health Services funded the MIRECC Initiative on Antipsychotic Monitoring Improvement (MIAMI) Project to broadly disseminate metabolic monitoring and management recommendations along with promising tools, strategies, and technical assistance to support relevant quality improvement efforts. The MIAMI Project team included clinical and research experts in psychopharmacology, SMI, and implementation science, who further operationalized metabolic monitoring and management recommendations. The MIAMI Project included:

1. A national training meeting (May 2010) attended by approximately 100 providers and clinical managers;

2. The MIAMI Project Technical Assistance Center (TAC) to assist providers and clinical managers in developing quality improvement efforts and to provide additional information on MIAMI Project tools/resources; and

3. A VA intranet website (http://vaww.mirecc.va.gov/miamiproject), with links to relevant clinical practice guidelines, educational resources, and weight management resources.

Available MIAMI Project tools include examples of computerized clinical reminders, a poster summarizing monitoring and management recommendations, an automated computer routine to identify patients due for initial metabolic monitoring, and a psychoeducational weight management program [27,28].

The protocol presented in this article is for the project, ‘Monitoring and Management for Metabolic Side Effects of Antipsychotics,’ which is testing an EBQI/F implementation intervention to enhance uptake of evidence-based tools and strategies to improve monitoring and management of metabolic side effects of antipsychotics concurrently with the MIAMI Project’s VA national implementation effort. We anticipate that the national implementation effort will improve care overall, although the improvement is likely to be variable as there are a substantial number of facilities that have fewer resources and more difficulty mounting QI efforts, and that do not have formal coordination between mental health and primary care clinics for managing metabolic side effects in patients taking antipsychotics. Therefore, we are studying the national implementation effort at multiple sites, using a cluster randomized design, assessing organizational challenges at these sites, and testing an intervention that includes EBQI [29,30] and external facilitation [31,32] strategies to customize, adapt and enhance the impact of local strategies for implementation of the national QI initiative. We stratified sites based on scores on an organizational practice survey, and randomized at the site level, recognizing that organizational context is likely to be a strong predictor of implementation success. Results will inform ongoing VA implementation of tools and strategies to improve metabolic monitoring and management, as well as future implementation research to improve care at all VA facilities, not just sites that have adequate resources and QI infrastructure.

### Study objectives

1. To test the effect of an EBQI/F intervention as an augmentation to the national implementation initiative on monitoring of metabolic side effects of antipsychotics in sites likely to encounter greater challenges to implementation (see Site Selection below).

2. To test the effect of the EBQI/F intervention as an augmentation to the national implementation initiative on management of metabolic side effects of antipsychotics in sites likely to encounter greater challenges to implementation.

3. To assess the direct costs of the EBQI/F intervention, and explore potential variations in costs of the EBQI/F intervention in sites with lower versus higher levels of organizational challenges.

## Methods

**Figure 1:**
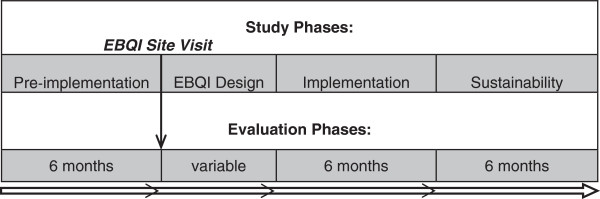
Study timeline of implementation/evaluation phases for each matched pair of sites.

This study employs a cluster randomized clinical trial design, with matched pairs of VA facilities defined as the clusters, to test the effectiveness of an EBQI plus facilitation (EBQI/F) intervention combined with the ongoing national quality improvement initiative (MIAMI Project) at six sites (intervention sites) compared to six matched comparison sites exposed to the national quality improvement initiative alone (control sites). The extent to which metabolic monitoring and management recommendations are followed will be examined at the intervention sites over three six-month intervals: the Pre-implementation Phase, defined as the six-month period prior to the initial EBQI site visit at the intervention sites; the Implementation Phase, which includes active external facilitation (EBQI/F); and the Sustainability Phase, during which the research team continues to evaluate the extent of metabolic monitoring and management, but external facilitation is not provided (see Figure 1). Each control site will be examined over the same three six-month periods as the matched intervention site. During the initial site visit, and for a variable period of time following the visit (the EBQI Design Phase), each intervention site’s EBQI participants will work with the facilitator to develop a local implementation plan. If preliminary results indicate that the EBQI/F intervention is successful, control sites will be offered the EBQI/F intervention in a Crossover Phase at the conclusion of the study.

### Site selection

Our systematic site selection method used data extracted from national VA databases and determined that 99 VA Medical Centers had 300 or more patients with a new antipsychotic prescription in the first six months of FY08. Next, to identify sites likely to perform relatively poorly in management of metabolic side effects, we determined the proportion of patients at each site who received a primary or secondary stop code for an encounter with the VA’s ‘MOVE!’ Weight Management Program in the six months following the new antipsychotic prescription. We used the referral to the ‘MOVE!’ Program as an indicator of provider attention to adverse medication side effects, since weight gain is a common side effect of antipsychotic use. The median proportion of patient encounters with the ‘MOVE!’ Program per site was 1.2% and the range was 0% to 4.3%. These results are similar to a previous finding that only 2% of overweight patients with schizophrenia receive recommended medication changes [33].

We then excluded sites not involved in research, using the Office of Research & Development’s directory of research programs. From those sites located within the contiguous 48 states with active research programs, we contacted the mental health clinical manager at the 24 sites with the lowest proportions of ‘MOVE!’ Program utilization (range 0% to 0.66%). Three of the 24 sites were excluded because of an active or planned research project to study metabolic monitoring and/or management. Two sites were excluded because they already had primary care clinicians integrated into mental health clinics. Two sites declined to participate, and leaders at three other sites did not respond to repeated requests for participation in the project. Leaders at the remaining 14 sites attended conference calls with the study PI to obtain further information, and each agreed to have their site included in the final site selection pool. Each completed a brief survey, adapted from a VA Clinical Practice Organizational Survey that included questions on provider stress and ability to pursue practice change [34,35]. Survey scores ranged from 16.1 to 74.2 on a 100-point scale, with higher scores indicating higher levels of stress and lower capacity to pursue practice change. Sites were ranked according to survey scores. The 12 sites with the highest scores were invited to participate in the study, and all accepted. We used the ranked list of sites to identify matched pairs with comparable survey scores, and then randomly selected one site from each pair to receive the intervention, using a random number generator tool available at http://www.random.org. The mean survey score of the intervention sites was 48.9 compared to 51.1 for the control sites. Shortly after this matching, two of the sites withdrew from study participation; however, fortunately, the remaining two sites that had not initially been selected for study inclusion agreed to participate. Both of these sites had low scores on the Clinical Practice Organizational Survey, and thus did not affect the distribution of intervention and control sites on this inclusionary variable. Soon after this change, another site withdrew from the study; however, this occurred at about the same time that one of the previous sites that had earlier declined participation was now willing to participate. Both of these sites had been randomized to the intervention arm, and so the newly available site replaced the withdrawn site, resulting again in 12 sites participating in the study: 6 intervention sites and 6 control sites.

### Participant selection and recruitment

Study participants include VA employees who are involved in the monitoring and management of patients treated with antipsychotics at participating sites and who completed surveys and interviews about related practices and quality improvement efforts; and VA patients prescribed antipsychotic medications, whose administrative and medical record data are obtained and analyzed to assess metabolic monitoring and management rates throughout the study. Employee participants were recruited solely by the research team to ensure confidentiality and avoid coercion. The study was reviewed and approved by the VA Central IRB and the Central Arkansas Veterans Healthcare System Research & Development Committee.

### EBQI/F intervention

Promoting Action on Research Implementation in Health Services (PARIHS) is the conceptual framework that guides and informs our intervention and evaluation approaches for this study. In this framework, successful implementation (SI) is represented as a function (f) of the nature and type of evidence (E), the qualities of the context (C) in which the evidence is being introduced, and the way the process is facilitated (F): SI = f(E, C, F) [36]. ‘Evidence’ includes knowledge obtained through research, clinician experiences, performance feedback, patient preferences, and other sources. ‘Context’ includes factors that may affect implementation of evidence-based practices, such as organizational culture/climate, readiness-to-change, leadership quality, resources, and the organization’s approach to evaluation. ‘Facilitation’ of the intervention by external and/or internal change agents can also affect implementation success [36,37].

The EBQI/F intervention, which involves researchers partnering with clinical stakeholders, is designed in a manner that is consistent with available research evidence and reflects the knowledge and expertise of site participants regarding how to tailor the strategy to local needs, priorities and resources. The intervention combines: EBQI processes in the EBQI Design Phase to design and tailor the initial implementation strategy to the local context, and external facilitation to support, monitor, problem-solve and refine implementation (as needed) during the study’s Implementation Phase. Further, through combining strategies and tools included in the MIAMI Project (see above) with EBQI and facilitation, the EBQI/F intervention provides a multifaceted approach to improving care in this area [38-41].

For the current project, the automated computer routine that was developed during the ASSIST Project (see above) was revised to identify patients who had been prescribed a new antipsychotic medication and were due for baseline or initial follow-up monitoring. Reports generated by this routine list weight, BMI, and relevant laboratory test results, if these tests were done. Additional computer routines were developed to generate a list of patients with abnormal laboratory results, and indicate whether management actions had been taken (*e.g*., prescription of a hypoglycemic agent following an elevated glucose test). The research team obtained approval from the VA Office of Information & Technology to install this software at study sites.

### EBQI design phase

The EBQI Design Phase includes an initial site visit and subsequent development of a local implementation plan at each site. In this phase, researchers contribute knowledge about the evidence base for clinical care and implementation strategies, providing implementation tools and materials. Clinical stakeholders contribute local knowledge and expertise needed to tailor implementation tools/strategies to match organizational needs, priorities and capabilities.

EBQI participants at each intervention site include approximately two to four key staff (*i.e*., administrative leaders, clinicians, nurses) involved in monitoring and managing patients treated with antipsychotics who were identified based on discussions between the study PI and site mental health leaders.

The EBQI Design Phase includes consideration of site-specific data collected in the developmental formative evaluation (see below), such as current practices, barriers/facilitators, key issues, and potential solutions in addressing project goals. These data were gathered through qualitative interviews conducted prior to the start of implementation activities, analyzed using a rapid analysis approach [42,43], and shared with EBQI participants to assist in development of local implementation strategies. In addition, EBQI participants were provided local performance data on metabolic monitoring and management, extracted from VA databases as described below for a six-month period prior to the site visit.

During the EBQI Design Phase, EBQI participants consider the QI tools/resources included in the MIAMI Project as well as additional monitoring and management tools and strategies that may be useful for local implementation. The duration of the EBQI Design Phase varies across sites, an understandable phenomenon in implementation studies, where preparation, design and implementation are often classified as distinct phases and differ depending on activities at each intervention site [44].

### Implementation phase: active facilitation

The Implementation Phase of this study is guided by an experienced and well-respected researcher (JLS), who serves as the lead facilitator, and a co-facilitator. Facilitator roles are to establish and leverage relationships with clinical stakeholders in order to encourage, support and guide them in executing the local implementation strategy.

It is expected that specific facilitation activities will vary by site because the context/needs of individual sites will also vary, while the roles of the facilitator will remain consistent with key components of external facilitation—including interactive problem-solving and support—as identified in our earlier MH QUERI research [37]. A time/activity log maintained by the facilitator will document all facilitation activities. This log will be used to estimate costs of the EBQI/F intervention as described in Data Analysis.

### Evaluation

Evaluation will include formative and summative evaluation activities. Formative evaluation (FE) is designed to identify potential and actual influences on the progress and effectiveness of implementation efforts. It also helps optimize the potential for success by providing a better understanding of the processes involved, identifying and addressing needed refinements to the implementation strategy, and assessing the merit of using similar approaches in future implementation efforts [45]. The FE for this project included developmental FE, implementation-focused FE, progress-focused FE, and interpretive evaluation. Summative evaluation will quantitatively assess the effectiveness of the EBQI/F intervention in comparison to sites randomized to the study’s control condition.

### Developmental FE

The objectives of developmental FE are to identify determinants of current practice regarding monitoring/management of metabolic side effects, identify available QI resources, identify barriers/facilitators, and engage clinical stakeholders in defining key issues and potential solutions in addressing project goals. Key staff at intervention and control sites have completed semi-structured telephone interviews, providing their perspectives on these issues. Participants also completed the Organizational Readiness to Change Assessment (ORCA), described below.

### Implementation- and progress-focused FE

The objectives of implementation-focused FE are to document implementation processes and exposure of site participants to the intervention, identify barriers to and deviations from the planned local implementation strategy, and modify the strategy as needed to overcome barriers [45]. The objective of progress-focused FE is to monitor progress in achieving implementation goals and performance targets, enabling the facilitator to modify intervention tools/strategies as needed to maximize potential for success [45]. The facilitator monitors implementation processes and progress through regular contacts with a primary contact at each intervention site. Because the facilitator’s attention to implementation- and progress-focused FE activities will be directed exclusively to EBQI/F sites, a survey of metabolic monitoring and management practices (SMM) will be administered to Mental Health Service Chiefs at all sites prior to the start of the Implementation Phase and at the end of the Sustainability Phase.

### Interpretive evaluation

The objective of interpretive evaluation is to obtain stakeholder perspectives on the perceived value of the EBQI/F intervention, barriers and facilitators encountered, satisfaction with the implementation process, unintended consequences, and any needed refinements. Only intervention sites receive interpretive evaluation.

Stakeholder experiences with the intervention will be assessed after the Implementation Phase through semi-structured qualitative telephone interviews with the two most involved participants in implementation activities at each EBQI/F site. All interviews will be audio-taped and transcribed for qualitative data analyses.

### Summative evaluation

#### Data sources

Summative evaluation data sources include data extracted from VA databases; medical record abstraction; and surveys (SMM and the Organizational Readiness to Change Assessment [ORCA]) administered to the participants. The VA datasets contain patient demographics, inpatient and outpatient encounters and associated diagnoses, pharmacy and laboratory data, and vital signs. Patient data on metabolic side-effect monitoring and management will be collected on all Veterans meeting inclusionary criteria at each site, and these data will be analyzed and presented at the VA facility level.

#### Metabolic side-effect monitoring

Data will be extracted for all patients who were treated at a study site and were prescribed a new antipsychotic medication (defined as a ‘new antipsychotic start’ or the addition of, or switch to, an antipsychotic medication that had not been prescribed in the previous 180 days) [20] and who would be due for monitoring or management of metabolic side effects during any of the three six-month phases of the study. If monitoring of a metabolic parameter (weight, glucose or hemoglobin A1c, and low-density lipoprotein [LDL]) is documented as occurring within the window of 30 days before to 30 days after a new prescription, we defined this as completed ‘baseline’ monitoring. The recommended 90-day ‘follow-up’ monitoring is defined as monitoring of a metabolic parameter between 31 and 120 days following the new prescription date. For each study site, the proportion of patients due for monitoring that received monitoring at each time point for each parameter will be calculated and aggregated for each month in each study phase. All patients whose monitoring window ends within a given month will be included in the denominator to calculate that month’s (baseline or follow-up) monitoring rate.

#### Metabolic side-effect management

Performance of recommended management actions will be assessed for patients with evidence of obesity (BMI ≥30), hyperglycemia (plasma glucose ≥200 mg/dL), elevated hemoglobin A1c (≥6.5), or elevated LDL (≥160 mg/dL) at either the baseline or 90-day follow-up window, or weight gain (>5% gain from baseline) at the 90-day follow-up window. Because VA datasets do not contain a reliable indicator of whether a laboratory test was performed when the patient was in a fasting state, these thresholds for management are set at a higher level than indicated in practice guidelines (*e.g*., 126 mg/dL for glucose). However, diabetes practice guidelines indicate that two random glucose tests with results ≥200 mg/dL may be used to diagnose diabetes [17].

Medical records will be reviewed to better assess initiation of management actions for patients who are found to have abnormal results on any of the metabolic parameters. This will not only allow for confirmation of the accuracy of clinical management actions captured by the electronic datasets, but will also allow for the inclusion of additional information, such as documentation of decision-making regarding antipsychotic choice and of counseling on diet and exercise. Possible management actions and the data source from which they are derived (VA datasets or chart abstractions) are listed in Table 1. Monthly measures of a site’s rate of management for metabolic side effects for these patients will be constructed, combining data from the two sources, using the data abstracted from the medical record as the gold standard.

**Table 1:** Operationalization of measures for guideline-recommended metabolic management

**Parameter**	**Thresholds**	**Management elements**	**Operationalization**	**Data source**
**Weight**	BMI ≥30, weight gain >5%	Antipsychotic management	Switch to medication w/ lower propensity for weight gain	VA dataset
		Lifestyle modification	Counseling on diet, exercise, and behavior modification within 30 days, OR	Chart review
			Nutrition consult within 30 days, OR	VA dataset
			‘MOVE!’ Program encounter(s) within 30 days	VA dataset
		Medication	Orlistat prescription within 30 days	VA dataset
**Glucose**	Plasma glucose ≥200 or Hgb A1c ≥6.5	Antipsychotic management	Switch to medication with lower propensity for elevated glucose	VA dataset
		Lifestyle modification	Counseling on diet, exercise, and behavior modification within 30 days, OR	Chart review
			Nutrition consult within 30 days, OR	VA dataset
			‘MOVE!’ Program encounter(s) within 30 days	VA dataset
		Follow-up	Primary care/diabetes clinic visit within 30 days	VA dataset
		Medication	Hypoglycemic agent within 30 days	VA dataset
**Lipids**	LDL ≥160	Antipsychotic management	Switch to medication w/ lower propensity for elevated lipids	VA dataset
		Lifestyle modification	Counseling on diet, exercise, and behavior modification within 30 days, OR	Chart review
			Nutrition consult within 30 days, OR	VA dataset
			‘MOVE!’ Program encounter(s) within 30 days	VA dataset
		Follow-up	Primary care/medicine visit within 30 days	VA dataset
		Medication	Statin prescription within 30 days	VA dataset

#### Data collection for and definition of covariates

A number of patient demographic and clinical characteristics could affect the frequency of vital sign determinations and laboratory tests for glucose or lipids. For example, patients with chronic heart failure should have weight recorded frequently; patients with diabetes would have more frequent fasting plasma glucose tests; and older patients and patients that have frequent primary care visits for any reason would also be more likely to meet criteria for recommended metabolic monitoring. Therefore, in addition to birth date, gender and race, we will extract diagnostic codes to identify pre-existing diagnoses of overweight/obesity, diabetes or dyslipidemia, as well as other physical comorbidities. These variables, summarized over site, will be included as covariates in the statistical models of monitoring and management.

#### Assessing organizational readiness-to-change

The effect of organizational readiness-to-change on implementation is being measured in this study with the Organizational Readiness to Change Assessment (ORCA) [46]. This instrument was developed for use in VA treatment settings, assessing organizational readiness in terms of PARIHS domains of evidence, context and facilitation, as well as the sub-elements of culture and leadership [47,48]. The 15- to 20-minute survey was administered to all provider participants at the 12 participating sites during the Pre-implementation Phase and will be repeated at the conclusion of the Sustainability Phase.

#### EBQI/F intervention costs

To estimate direct intervention costs (Objective 3), we adapted the methodology used in the Translating Initiatives in Depression into Effective Solutions (TIDES) study [44]. We will explore potential variations in intervention costs at sites with lower versus higher organizational readiness-to-change. We will assign costs to one of three categories of implementation activities, as shown in Table 2.

**Table 2:** Intervention costs for EBQI/F implementation activities

**Activity category**	**Examples of activities included**
**Implementation design**	In-person EBQI design meeting
	Introductory educational conferences by experts on research team
	Meeting preparation
**Clinical informatics**	Preparation of site feedback reports
	Customization of VA computer routines based on EBQI/F site visit requests
	Installation, pilot testing, and refinement of VA computer routines
**Facilitation**	External facilitation activities, including communication with site participants, coordinating technical assistance, troubleshooting, and problem-solving

### Data analysis

Because weight gain is a common antipsychotic side effect that increases risk of diabetes and cardiovascular disease [6,9,11], and overweight/obesity is prevalent in patients with psychotic disorders [10,11], the primary outcome measures for the proposed project will be the extent to which weight/BMI is monitored and to which overweight or obese patients receive a guideline-concordant weight management intervention. At each site, the proportion of patients who receive baseline monitoring, follow-up monitoring, and management of metabolic abnormalities will be measured each month against those who were due for such actions during the same time period. We expect EBQI/F intervention sites to gradually increase performance on monitoring and management measures over time. Therefore, we will conduct a time series analysis of monthly performance, with each site contributing six monthly performance measures in each of the three phases of the study. These analyses will estimate the change in rates of baseline monitoring during the Implementation Phase due to intervention (national quality improvement initiative with and without EBQI/F), and allow comparison of these performance changes between sites receiving EBQI/F and those not. Further, these analyses will allow within-phase changes in monitoring rates to be compared between phases; *e.g*., for EBQI/F sites, is the monitoring performance change during the Implementation Phase different from that during the Sustainability Phase? We will similarly collect, summarize, and analyze data from all patients due for follow-up metabolic monitoring and metabolic management (as defined in Table 1) during the three study phases.

We will compare the performance change over time (a slope) between EBQI/F and control sites using a random coefficients regression (RCR) that accounts for repeated measures taken within a site (see Additional file 1 for detailed explanation). The random coefficients portion of the RCR accounts for site-to-site variability within an intervention group (*e.g*., EBQI/F); that is, each site has its own slope, but sites within the same intervention group tend to be more alike than those from the other intervention group. The RCR will also allow the slopes to change with the phase of the study, thus allowing us to also test whether performance change differs among the phases over the study. For example, performance change during pre-implementation is expected to be unchanging or flat, but is expected to increase or trend upward once EBQI/F begins.

Descriptive analysis of intervention costs will include calculation of the number of providers involved in each phase of the EBQI/F intervention (person count) and the number of hours spent (person hours), with separate estimates for clinical QI and research team activities. We will also explore whether there may be potentially meaningful variations in costs/intensity of the EBQI/F intervention at the EBQI sites based on their readiness-to-change, using data collected from the ORCA.

### Power analysis

Based on our six EBQI/F intervention sites and six control sites, Table 3 presents detectable slopes (*i.e*., within-phase performance change) and changes in slopes (between phases) assuming first: low, medium and high values for the residual standard deviation (σ), the variances of the random intercepts (*v*_*a*_) and slopes (*v*_*b*_), and a first order auto correlation, ρ, among the observations within a site; then, power of at least 0.8 on a two-sided test made at the 0.05 significance level. Using ASSIST study findings, we estimated residual standard deviations (σ) ranged from 8.9% to 11.9%, *v*_*a*_ from 53.4 to 167, *v*_*b*_ from 4.4 to 6.6, and within-site first order auto correlations (ρ) from 0.10 to 0.53. We used the minimum, midrange, and maximum of the estimates to respectively present best case (σ = 8.9%, *v*_*a*_ = 53.4, *v*_*b*_ = 4.4, ρ = 0.10), expected (σ = 10.1%, *v*_*a*_ = 137.6, *v*_*b*_ = 5.3, ρ = 0.26), and worst case (σ = 11.9%, *v*_*a*_ = 167.0, *v*_*b*_ = 6.6, ρ = 0.53), scenarios. Given the low rates of metabolic monitoring and management observed in preliminary studies, the detectable changes would be both possible and clinically meaningful.

**Table 3:** Detectable differences in slope (% change per month)

**Hypotheses**	**Best case scenario**	**Expected scenario**	**Worst case scenario**
**Implementation has no effect over phase**	3.7%	4.3%	5.1%
**Implementation is no different from control at each phase**	5.3%	6.1%	7.2%

### Trial status

The study is in the initial part of the Implementation Phase. The initial EBQI site visits to the six implementation sites were completed in 2012, and facilitation activities have begun at the intervention sites. Retrospective data are now being collected from all study sites.

## Discussion

This study will build on prior research, demonstrating the effectiveness of EBQI and facilitation to support tailored implementation efforts at a site, and addresses an important aspect of clinical care for patients taking antipsychotics. Improving monitoring for metabolic side effects of antipsychotics, as well as promoting timely evidence-based management when these effects emerge, will lead to improved patient safety and long-term outcomes. This project aims to increase uptake of the existing tools and strategies that have been made available through a national dissemination effort, although the facilitation process could result in refinements of these elements or in development of new improvement approaches. We discuss strengths and initial challenges of the study below.

### Site selection

The research team employed a systematic site selection process rather than identifying a convenience sample of frequently-utilized study sites. The selected sites thus are more likely to be representative of the diversity of VA facilities, with respect to size, extent of academic affiliation, resources available to pursue quality improvement efforts, and scores on the organizational practice survey than would have been the case if a convenience sample of sites was selected. Systematic site selection ensures that study findings will be broadly generalizable to the majority of VA facilities, and enables the research team to identify unique site implementation barriers and facilitators.

Despite the potential benefits of this site selection method, it also has caused challenges. While we pursued IRB approval, our contacts at 3 of the 12 initially selected sites indicated that the site would be unable to participate in the study (*e.g*., because the leader who originally agreed was no longer in the same position). This experience suggests that a disadvantage of the systematic site selection process is that there may be more attrition than when convenience sampling is employed; perhaps investigators could develop retention strategies to maintain site leaders’ interest in the study and encourage them to overcome participation barriers during the sometimes lengthy waiting period between grant submission and final funding. A study team could include back-up sites in the site selection approach, although it may not be possible to activate a replacement site once the study has started. Finally, investigators should recognize that a site that withdraws after the start of implementation activities should still be included in an ‘intent-to-implement’ analysis.

### Timing of EBQI/F intervention

The grant application was written in 2008, with the expectation that the EBQI/F intervention could be initiated concurrently with the planned initiation of the MIAMI Project dissemination and implementation activities. The national training for the MIAMI Project occurred in 2010. However, due to the time elapsed between grant submission and IRB approval, and a subsequent delay related to obtaining data access and approval for use of our automated computer routines, the study intervention site visits did not begin until late 2011, more than a year following the start of MIAMI Project activities. Therefore, the project is studying the added effect of EBQI/F on top of an ongoing dissemination effort, rather than a test of the effectiveness of EBQI/F delivered at the time that a national implementation activity was rolled out, as was originally envisioned. This means that during the ‘Pre-implementation Phase,’ both intervention and control sites have MIAMI Project resources available, rather than this period representing a baseline period prior to MIAMI Project roll-out. However, relatively few providers avail themselves of the educational information, computer routines, or support services. Thus, we expect that the EBQI/F intervention could result in substantial improvement in monitoring and management practices.

### Definition of abnormal ranges for glucose and LDL

Evidence- and consensus-based guidelines recommend obtaining plasma glucose and lipid tests in the fasting state, and intervening if test results are elevated (*e.g*., glucose ≥126 mg/dL; LDL ≥130 mg/dL) [17,18]. Unfortunately, neither VA databases nor the narrative electronic medical record reliably indicate whether a patient was fasting at the time of laboratory testing [20]. Therefore, our study uses higher cut-off points (glucose ≥200 mg/dL; LDL ≥160 mg/dL) in order to increase the likelihood that elevated glucose or LDL are, in fact, indicative of diabetes or dyslipidemia respectively and warrant management action(s), rather than being an artifact of the non-fasting state. Other methods could be used to determine whether the patient was likely to be fasting, such as the length of time between the clinical visit and the tests; the administration of glucose and lipid assays on the same day, which could indicate that the tests were intended to be drawn in the fasting state [49]; or examining chart documentation for evidence that fasting was recommended prior to testing. However, this information may be incomplete and may vary among providers and sites. In contrast, using the higher cut-offs for Objective 2 analyses will increase the likelihood that abnormal glucose or LDL tests are truly abnormal and require some form of management. This method reduces false positives and increases specificity at the cost of reduced sensitivity.

We also chose to focus our Objective 2 examination of management actions on those patients whose testing indicated obesity (BMI ≥30) or weight gain (>5%), rather than including the larger group of patients who warrant management for being overweight (BMI between 25 and 30). It would not be feasible to review the medical records for all patients with BMI greater than or equal to 25. Excluding these patients, as well as patients with possible abnormal glucose or LDL values, represents a limitation in the study methods. We will further explore this issue by conducting a sensitivity analysis by assessing the extent to which side-effect management takes place for a subsample of patients who are overweight, have blood glucose between 126 and 200 mg/dL, or have LDL between 130 and 160 mg/dL. We will also explore whether chart documentation indicates that a given blood test was meant to be obtained while the patient was fasting. These sensitivity analyses could provide useful information for future studies and could be used to encourage inclusion of fasting notations in the VA databases.

Despite the challenges and limitations of this complex study of implementation strategies, this ongoing study will yield insights into barriers and facilitators to monitoring and management of antipsychotic side effects; determine the effectiveness of EBQI combined with external facilitation with regard to promoting successful implementation; and hopefully will improve care and patient safety at the intervention sites. Study findings will inform subsequent VA efforts to improve care and safety for patients prescribed antipsychotic medications.

## Abbreviations

ASSIST: A study of strategies for improving schizophrenia treatment; ADA: American diabetes association; APA: American psychiatric association; BMI: Body mass index; CATIE: Clinical antipsychotic trials of intervention effectiveness; CPG: Clinical practice guidelines; DoD: Department of defense; EQUIP: Enhancing quality and utilization in psychosis; EBQI/F: Evidence-based quality improvement with facilitation; FPG: Fasting plasma glucose; FE: Formative evaluation; LDL: Low-density lipoprotein; MH QUERI: Mental health quality enhancement research initiative; MIRECC: Mental illness research, education, and clinical center; MIAMI: MIRECC initiative on antipsychotic monitoring improvement; OIG: Office of inspector general; OL: Opinion leader; PARIHS: Promoting action on research implementation in health services; SGA: Second-generation antipsychotic; SMI: Serious mental illness; SMM: Survey on metabolic side-effect monitoring and management; VistA: Veterans health information systems and technology architecture; VISN: Veterans integrated service network.

## Competing interests

The authors declare that they have no competing interests.

## Authors’ contributions

All authors have made substantial contributions to the study conception and design, as well as to the development and editing of the manuscript. RO and JS led the initial study design and development and wrote the original study protocol. RL planned the statistical analyses and performed the power analysis. KD, KM, and SP assisted with further refinement of the study protocol. RO and SP drafted the publication with contributions from all authors. All authors are contributing to the conduct of the study and have read and approved the manuscript for publication.

## Supplementary Material

Additional file 1 Detailed Description of Data Analysis Methods for Objectives 1 and 2 Time Series Analysis. Click here for file
