# Supplementary material for: Monitoring and managing metabolic effects of antipsychotics: a cluster randomized trial of an intervention combining evidence-based quality improvement and external facilitation
Source: Implement Sci. 2013 Oct 8;8:120. doi: 10.1186/1748-5908-8-120 (PMC3852845; doi:10.1186/1748-5908-8-120)
Supplement: Additional file 1 — Detailed Description of Data Analysis Methods for Objectives 1 and 2 Time Series Analysis. [file 1748-5908-8-120-S1.pdf]

Additional File 1. Detailed Description of Data Analysis Methods for Objectives 1 and 2

Time Series Analysis: The primary outcomes of interest are performance on weight monitoring and management measures over the pre-implementation, implementation, and sustainability phases of the study. At each site, monthly performance will be measured as proportions of patients who receive baseline or follow-up monitoring or management of metabolic abnormalities among those who are due for such actions during the same time period. We expect that the impact of the Evidence-Based Quality Improvement/Facilitation (EBQI/F) intervention will be realized gradually, increasing performance on monitoring and management measures over time. Therefore, we will conduct a time series analysis of monthly performance. Each site will contribute six monthly performance measures in each of the three phases of the study. These measures will be modeled with a random coefficients regression that allows for the slopes associated with time to change at each phase for both the study conditions (EBQI/F and control) and random sites. To allow modeling of different slopes within each study phase, we will include indicator variables for each of the three phases. (This is sometimes referred to as a "change point regression.") Specifically, the proposed model for the expected outcome, E(Y), is:

$$
\begin{aligned}
E(Y_{jkl}) = \; & P1*[\,(\alpha + a_{jk}) + (\beta_{1j} + b_{1jk})X_l\,] \\
& + P2*[\,(\beta_{1j} + b_{1jk})X_{P2\ start} + (\beta_{2j} + b_{2jk})X_l\,] \\
& + P3*[\,(\beta_{2j} + b_{2jk})X_{P3\ start} + (\beta_{3j} + b_{3jk})X_l\,]
\end{aligned}
$$

where j, k and l, respectively, represent intervention condition, site within intervention condition, and observation within site. P1, P2, and P3 indicate pre-implementation, implementation and sustainability phases, respectively. Intercepts and slopes for intervention conditions are denoted with α and β, respectively, and site intercepts and slopes with "*a*" and "*b*", with subscripts 1 – 3 on β and b indicating the respective study phases. We note that at the

beginning of data collection (six months prior to the beginning of the implementation phase) all sites have the same expected intercept and slope, (α, β_1) due to randomization. We also point out that the intercepts for the second and third phases begin at the endpoint of the first and second phases, respectively; thus the $(\beta_1 + b_{1jk})X_{P2\ start}$ and $(\beta_{2j} + b_{2jk})X_{P3\ start}$ terms. Site random effects, $(a, b_1, b_2, b_3)_{jk}$, will be modeled as multivariate normal with a zero mean vector and diagonal covariance matrix. We will account for the intra-site correlation (i.e., repeated measures or time series nature of the data) in our model for Var(Y_{jk}). Sensible models for Var(Y_{jk}) are first order autoregressive, compound symmetric, and Toeplitz covariance structures. The covariance model having the best Bayesian Information Criterion (BIC) will be used. We will also examine whether there is evidence that the distributions of patient-level covariates, summarized each month at each site as means or proportions, including age, gender, race, pre-existing diagnoses of overweight/obesity, diabetes, or dyslipidemia, and other physical health comorbidities (see above), covary with the site-level monthly proportions of monitoring or management. If there is evidence that any of these covariates have a relationship with the site-level outcome variables, we will enter these into the model described above. Due to the relatively few sites from which data will be collected, however, the power to detect effects of these covariates will likely be reduced from the projected power described below.

The model associated with the equation above will be used to address the hypotheses in Objectives 1 and 2 (see below). To determine whether the control condition (exposure to national implementation initiative alone) (j = 1) and the EBQI/F intervention (j = 2) significantly improve measures of metabolic monitoring and management, we will test whether the corresponding slopes during the implementation phase (β_{21} and β_{22}) are equal to zero (Hypotheses 1a, 2a, and 3a; see Figure 1). We will then compare slopes of monitoring/management measures for the EBQI/F and the control conditions during the

implementation phase to determine whether $\beta_{21} = \beta_{22}$ (Hypotheses 1b, 2b, and 3c). Finally, we will examine intervention sustainability by testing whether $\beta_{31} = \beta_{32}$ (Hypotheses 1c, 2c, and 3c).

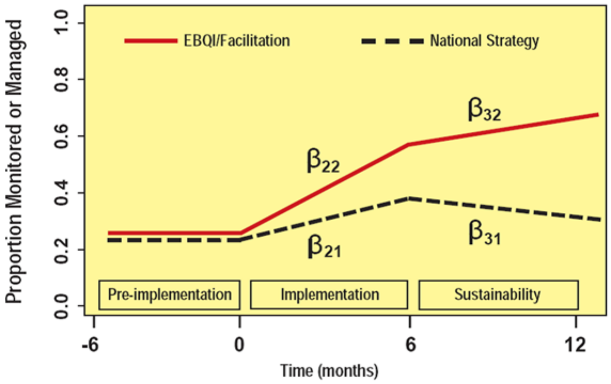

Figure 1. Hypothetical Time Trends

Objective 1: To test the effect of an EBQI/F intervention as an augmentation to the national implementation initiative on *monitoring* of metabolic side effects of antipsychotics in sites likely to encounter greater challenges to implementation.

Hypothesis 1a: Rates of metabolic monitoring (weight/body mass index, glucose, low-density lipoprotein [LDL]) at the time of a new antipsychotic prescription (baseline) and 1-4 months later (follow-up) will improve significantly at EBQI/F intervention sites.

Hypothesis 1b: During the implementation phase, improvement in rates of metabolic monitoring will be significantly greater at EBQI/F sites than at sites assigned to the control condition.

Hypothesis 1c: During the sustainability phase, improvements in rates of metabolic monitoring at EBQI/F sites will be more likely to be sustained than will improvements at sites assigned to the control condition.

All patients due for *baseline* metabolic monitoring during the three study phases will be identified. A dichotomous variable for each metabolic parameter will indicate whether baseline monitoring was performed within 30 days of a new antipsychotic prescription. Patient-level data will then be aggregated monthly into site-level data. A time series analysis of the site-level data will be conducted as described above to compare the effect of the EBQI/F intervention on baseline monitoring to the effect of the national implementation strategy alone and to determine

if monitoring performance during the implementation phase is different than during pre-implementation or sustainability phases. We will similarly collect, summarize, and analyze data from all patients due for *follow-up* metabolic monitoring during the three study phases.

Objective 2: To test the effect of the EBQI/F intervention as an augmentation to the national implementation initiative on *management* of metabolic side effects of antipsychotics in sites likely to encounter greater challenges to implementation.

Hypothesis 2a: Rates of guideline-concordant weight management will improve significantly at EBQI/F intervention sites.

Hypothesis 2b: During the implementation phase, improvement in guideline-concordant weight management will be significantly greater at EBQI/F sites than at sites assigned to the control condition.

Hypothesis 2c: During the sustainability phase, improvements in rates of weight management at EBQI/F sites will be more likely to be sustained than will improvements at sites assigned to the control condition.

Operationalization of management actions for overweight, obesity or weight gain, and data sources for evaluating performance of these management actions, are described elsewhere. The proportion of patients meeting criteria for weight gain, overweight or obesity who receive recommended weight management within 30 days will be computed monthly for each site. As described above, we will compare site-level weight management practices in pre-implementation, implementation, and sustainability phases, using time series analysis.

Hypothesis 3a: Rates of guideline-concordant assessment and primary care referral for elevated glucose or LDL will improve significantly at EBQI/F intervention sites.

Hypothesis 3b: During the implementation phase, improvement in rates of guideline-concordant assessment and primary care referral for elevated glucose or LDL will be significantly greater at EBQI/F sites than at sites assigned to the control condition.

Hypothesis 3c: During the sustainability phase, improvements in rates of guideline-concordant management of elevated glucose/LDL at EBQI/F sites will more likely be sustained than will improvements at sites assigned to the control condition.

Operationalization of management actions for elevated glucose and LDL, and data sources for evaluating performance of these management actions, are described elsewhere. The proportion of patients with elevated glucose and/or LDL levels who have primary care encounters within 30 days of the abnormal test will be collected from each site on a monthly basis. The monthly site-level data will then be subjected to the time-series analysis described above in order to evaluate the evidence in support of Hypotheses 3 and 3a. In addition, chart review data for patients with elevated values will be analyzed to estimate the extent to which providers documented that they encouraged the patient to have a primary care appointment, but the referral was not completed successfully.
